# Recurrence of Diffuse Alveolar Hemorrhage via Postoperative Thrombotic Valve Formation after Mitral Valve Bioprosthetic Replacement

**DOI:** 10.7759/cureus.84313

**Published:** 2025-05-18

**Authors:** Ryo Nakada, Daisuke Ueshima, Maki Ono, Toshikazu Kono, Akira Mizukami

**Affiliations:** 1 Cardiology, Kameda General Hospital, Kamogawa-shi, JPN

**Keywords:** antiphospholipid antibody syndrome, bioprosthetic valve replacement, diffuse alveolar hemorrhage, mitral valve regurgitation, mitral valve stenosis, systemic lupus erythematosus, thrombotic valve

## Abstract

A 39-year-old woman who had been receiving steroid treatments for systemic lupus erythematosus (SLE) and antiphospholipid antibody syndrome (APS) had 11 emergency hospitalizations over 18 months for diffuse alveolar hemorrhage (DAH). She had severe mitral stenosis and regurgitation, so mitral valve replacement with a bioprosthetic valve was performed in November 2022. There was no recurrence of DAH until May 2023 (six months after the surgery). However, her mitral stenosis worsened, and DAH recurred following postoperative thrombotic valve formation. Inadequate postoperative antithrombotic therapy might have led to the formation of a thrombotic valve; however, we were able to improve the patient’s mitral stenosis and thus prevent the recurrence of DAH through antithrombotic therapy reinforcement. In conclusion, we considered that not only SLE/APS-related immune-mediated capillaritis but also chronic increased pulmonary capillary pressure and left atrial pressure caused by mitral valve disease may have promoted DAH. Additionally, strict and long-term antithrombotic therapy is particularly important in patients with APS who undergo bioprosthetic valve replacement procedures.

## Introduction

The prevalence of diffuse alveolar hemorrhage (DAH) associated with systemic lupus erythematosus (SLE) is only from 0.5% to 5.4% [1,2]. However, the mortality rate in patients with the condition who experience DAH ranges from 40 to 50% [1]. This makes it one of the fatal complications of SLE. The exact etiology is still unclear, but DAH is thought to occur when the alveolar-capillary membrane is damaged. This damage may result from a sudden increase in pulmonary capillary pressure, direct injury, or inflammation, leading to rupture and subsequent bleeding into the alveolar spaces [3-5]. here are many reasons for it: small vessel vasculitis; connective tissue disease; heart diseases such as mitral stenosis, left ventricular dysfunction, and atrial myxoma; toxins; and infection [6]. In the case of mitral stenosis, increased left atrial pressure leads to elevated pulmonary capillary pressure, which can result in alveolar hemorrhage [7]. But there have been few reports about DAH caused by mitral regurgitation [8,9]. There have also been no reports in the literature thus far showing that surgical treatment improves DAH in this patient group.

And antiphospholipid antibody syndrome (APS) is an autoimmune disease wherein circulating antiphospholipid antibodies in the blood cause recurrent arteriovenous thrombosis [10]. There have been some reports on the formation of thrombotic valves following mechanical mitral valve replacement in patients with APS [11,12]. However, there are few reports specifically addressing cases involving bioprosthetic mitral valve replacement [13].

We report one case of frequent DAH recurrence that was prevented by mitral valve bioprosthetic replacement but recurred via postoperative thrombotic valve formation.

## Case presentation

**Figure 1:**
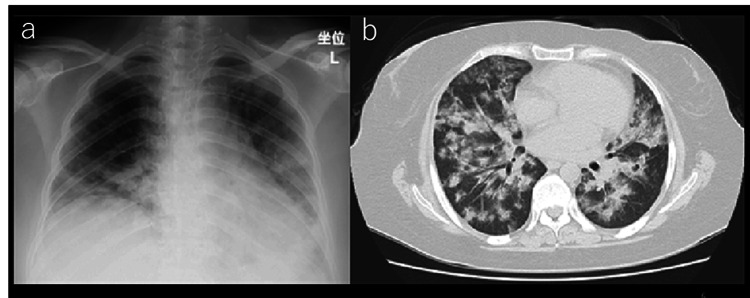
Chest radiography (a) and CT scan (b) following each occurrence of DAH DAH: Diffuse alveolar hemorrhage

Our patient was a 39-year-old woman who had been receiving steroid treatment for SLE and APS. She had also been diagnosed with moderate mitral regurgitation and severe mitral stenosis in 2021. As she was receiving high doses of steroids (prednisolone, 80 mg) and had severe obesity (BMI 43 kg/m^2^), the risk associated with surgical treatment was considered very high; therefore, the initial treatments focused on risk management. Over time, her steroid dose and BMI were gradually lowered to 5 mg and 30 kg/m^2^, respectively; however, the patient was hospitalized for DAH 11 times over a span of 18 months. The patient’s chest radiography and CT data at the time of 11th hospitalization are presented in Figure 1.

**Figure 2:**
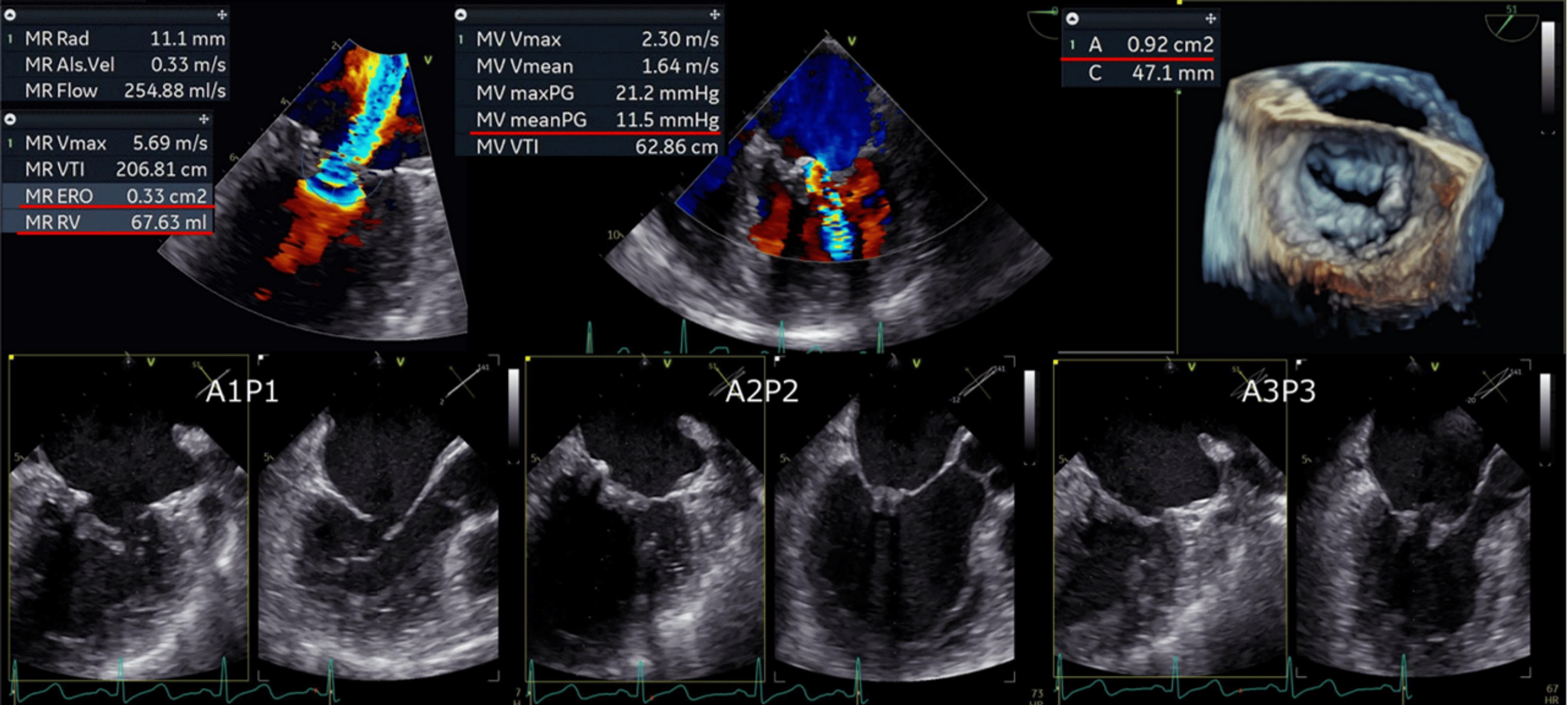
Transesophageal echocardiography data during the occurrence of DAH DAH: Diffuse alveolar hemorrhage

The patient’s transesophageal echocardiography data during the 11th hospitalization are presented in Figure 2. Her mitral valve leaflets were thickened and had calcification. The pressure gradient between the left atrium and left ventricle was 11.5 mmHg, and her mitral valve area (as surveyed via planimetry) was 0.92 cm^2^. Her effective mitral regurgitant orifice was 0.33 cm^2^, and the volume of mitral regurgitant was 67.6 ml (heart rate (HR) 90bpm). So, her mitral valve disease had progressed to severe mitral regurgitation and severe mitral stenosis. Additionally, her E/e′ ratio was 35. The results of the right heart catheterization performed after the acute treatment of DAH are as follows: pulmonary capillary wedge pressure: 16 mmHg; pulmonary artery pressure: 32/15/21 mmHg (systolic/diastolic/mean); right ventricular end-diastolic pressure: 8 mmHg; central venous pressure: 8 mmHg; Fick cardiac output: 3.7 L/min; Fick cardiac index: 2.0 L/min/m²; pulmonary vascular resistance: 108 dynes/sec/cm⁻⁵; systemic vascular resistance: 1,742 dynes/sec/cm⁻⁵.

**Figure 3:**
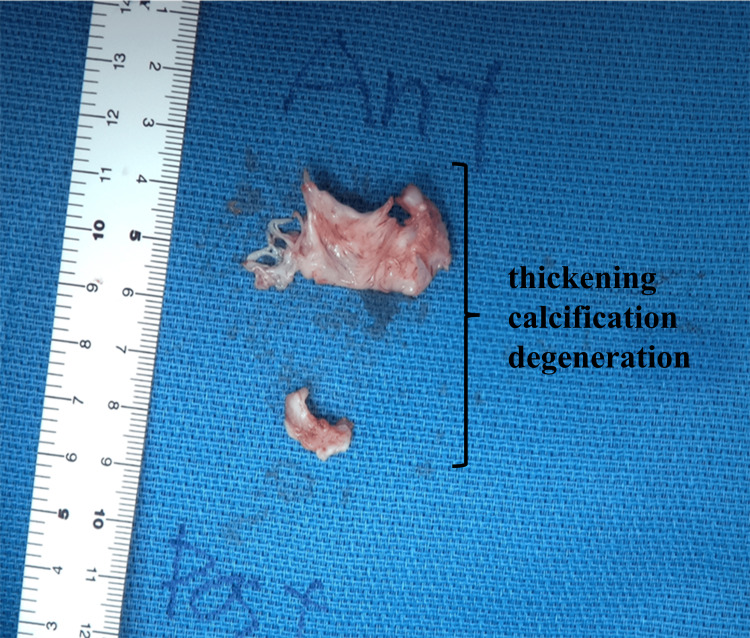
Pathological specimen of the mitral valve leaflet

The relationship between DAH and severe mitral valve disease was initially unclear, but pulmonary circulation disorder due to mitral valve disease was suspected. Therefore, the patient ultimately underwent mitral valve replacement with a 25-mm bioprosthetic valve (MITRIS), a product manufactured by Edwards (Irvine, USA), to treat both conditions in November 2022. Immediately after surgery, the pressure gradient between the left atrium and ventricle by transthoracic echocardiography was 3 mmHg (HR 81 bpm); severe mitral regurgitation and severe mitral stenosis was improved to trivial mitral regurgitation and mild mitral stenosis; and E/e′ ratio decreased from 35 to 14. Her mitral valve leaflet pathological findings included thickening, calcification, fresh blood clot attachment, and fibrinoid myxomatous degeneration (Figure 3). This histologically suggested Libman-Sacks endocarditis associated with SLE and APS. In general, if a mechanical valve had been chosen, the patient would need to use warfarin long-term to prevent thromboembolism. However, the patient expressed a desire to become pregnant in the future. Since warfarin carries risks of teratogenicity and fetal bleeding during pregnancy, we opted for a bioprosthetic valve instead to minimize long-term anticoagulation. Although warfarin was temporarily prescribed after surgery to prevent early valve thrombosis, our plan was to discontinue it before the patient attempts pregnancy.

**Figure 4:**
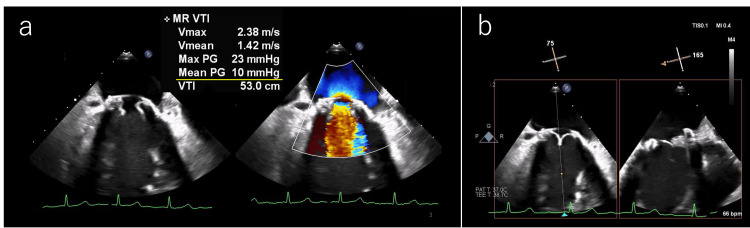
Follow-up transesophageal echocardiography performed seven months after the mitral valve replacement surgery; recurrence of severe mitral stenosis (a), and thrombotic valve formation and immune complex deposition (b)

Following the surgery, we maintained the patient’s prothrombin time-international normalized ratio (PT-INR) range between 1.6 and 2.0 to be careful not only of thrombosis but also bleeding. After the surgery, there had been no recurrence of DAH. We initially thought that the replacement had eliminated her frequent DAH; however, it eventually recurred postoperatively in May 2023 (six months after the surgery). On a follow-up transesophageal echocardiography, we found that the pressure gradient between her left atrium and ventricle was 10 mmHg, indicating severe mitral stenosis. We also noted the appearance of a certain structure when the two leaflets closed. One of the three bioprosthetic valve cusps had thickened and exhibited restricted movement. A mixture of high and low brightness was noted alongside the thickening, leading us to suspect thrombotic valve formation and immune complex deposition (Figure 4).

We concluded that the patient’s mitral stenosis had worsened, and that the mitral valve we observed was found to be thrombotic. We also suspected that the patient’s mitral valve disease was related to her episodes of DAH. Following consultation with surgeons, reoperation was one option. However, this case involved early recurrence, and the patient was considered too young (under specific age, e.g., 50 years old) to undergo reoperation at this time. We therefore elected to treat her via antithrombotic therapy instead. We considered that inadequate postoperative antithrombotic therapy might have led to the formation of the thrombotic valve, so we adjusted her PT-INR range from 2.5 to 3.5 using warfarin. After that, the pressure gradient between the left atrium and ventricle by transthoracic echocardiography in May 2025 was measured to be 5 mmHg (HR 76 bpm), so mitral stenosis has improved from severe to moderate. This approach successfully improved her mitral stenosis and prevented another recurrence of alveolar hemorrhage for 24 months. Changes in the pressure gradient between the left atrium and ventricle over time are summarized in Table 1.

**Table 1:** Progress of the pressure gradient between the left atrium and ventricle

Time Point	Pressure Gradient between Left Atrium and Ventricle
Immediately before surgery	11.5 mmHg
Immediately after surgery	3 mmHg
6 months after surgery (when DAH recurred postoperatively)	10 mmHg
24 months after strengthening of antithrombotic therapy	5 mmHg

## Discussion

DAH is one of the rare and deadly complications of SLE. In the present case, our patient’s frequent DAH, which was severe enough to require intubation, was successfully treated via mitral valve replacement. To the best of our knowledge, there have been no reports in the literature thus far showing that surgery improves alveolar hemorrhage. However, the relation between mitral valve replacement and DAH is nothing more than temporal and hypothetical, so we need further studies. Both mitral stenosis and mitral regurgitation increase left atrial pressure. There are several possible causes of DAH. In this case, we considered that her DAH may have been promoted not only by immune-mediated capillaritis related to SLE/APS, but also by chronically elevated pulmonary capillary and left atrial pressures resulting from mitral valve disease (including mitral stenosis and mitral regurgitation). Accurately assessing pulmonary capillary pressure and left atrial pressure is important in understanding the hemodynamic factors contributing to DAH, particularly when structural heart disease is suspected. Although pulmonary capillary pressure cannot be measured directly, it is estimated to lie between the systolic pulmonary arterial pressure and the pulmonary arterial wedge pressure [14]. In addition, the E/e′ ratio, which correlates with left atrial pressure, can be useful: an estimated left atrial pressure greater than 25 mmHg may be indicative of DAH [15]. Therefore, right heart catheterization and echocardiography are valuable tools for evaluating these pressures in patients with suspected DAH of cardiogenic origin. 

In this case, unfortunately, DAH occurred again because of the formation of the thrombotic valve. The frequency of thrombotic valve formation has been reported to range from 0.37 to 1.26% for bioprosthetic aortic valves and is approximately 6% for bioprosthetic mitral valves [13,16]. Few studies in literature have reported on thrombotic valve formation following bioprosthetic valve replacement in patients with APS. In Japanese guidelines, we should select surgical replacement when prosthetic valve dysfunction and heart failure symptoms occurs (Class Ⅰ). However, some reports suggest that the decision between surgical replacement and antithrombotic therapy should be made on a case-by-case basis, considering the capability of each facility [17]. In this case, the patient had early recurrence and was considered too young to undergo reoperation, so we selected antithrombotic therapy. Antiplatelet therapy with low-dose aspirin alongside antithrombotic therapy with warfarin, with a target PT-INR of from 2.0 to around 3.0, is generally recommended for patients with APS and thrombosis [18]. One notable report discussed a case wherein a thrombotic valve formed early during the postoperative period because of inadequate postoperative antithrombotic therapy, and antithrombotic therapy with warfarin was adjusted with a target PT-INR of 2-3 [19]. After surgery, we worried about bleeding by DAH and adjusted PT-INR range between 1.6 and 2.0. But thrombotic valve formation had already occurred, and we needed to prioritize the treatment of thrombosis. And, according to Japanese guidelines, warfarin control of INR 2.5-3.5 is reasonable for patients with thrombotic event despite adequate anticoagulation therapy (Class Ⅱa) [17]. Thus, we raised her PT-INR target range to lie between 2.5 and 3.5. This further emphasizes the importance of strict and long-term antithrombotic therapy in this patient group.

## Conclusions

DAH is one of the rare and deadly complications of SLE. In this case, we considered that it was promoted by not only SLE/APS-related immune-mediated capillaritis but also chronic increased pulmonary capillary pressure and left atrial pressure caused by mitral valve disease. The patient also experienced the formation of a thrombotic valve after bioprosthetic mitral valve replacement, and her mitral stenosis worsened. Strict and long-term antithrombotic therapy is particularly important in patients with APS who undergo bioprosthetic valve replacement procedures.
